# How breaking a sweat affects mood: The mediating role of self-efficacy between physical exercise and emotion regulation ability

**DOI:** 10.1371/journal.pone.0303694

**Published:** 2024-06-13

**Authors:** Fan-zheng Mu, Jun Liu, Hu Lou, Wei-dong Zhu, Zhen-cheng Wang, Bo Li

**Affiliations:** 1 Institute of Sports Science, Nantong University, Nantong, China; 2 School of Basic Medicine, Nanjing Medical University, Nanjing, China; The Open University of Israel, ISRAEL

## Abstract

**Objective:**

This study investigates the association between physical exercise and emotion regulation abilities among college students, introducing self-efficacy as a mediating variable to analyze the pathway mechanism through which physical exercise affects emotion regulation abilities.

**Methods:**

A cross-sectional study design was employed, utilizing a stratified random sampling method to survey three colleges in Jiangsu Province, China. Physical Activity Rating Scale, Physical Activity Self-efficacy Scale, and Emotional Intelligence Scale were used to measure the college student population. Regression analysis and mediation tests assessed whether self-efficacy mediates the relationship between physical exercise and college students’ emotion regulation abilities. A total of 5,430 valid questionnaires were collected.

**Results:**

The distribution of college students’ physical activities was 77.0% for low, 13.1% for medium, and 9.3% for high levels. Physical activities were significantly and positively correlated with self-efficacy and emotional management abilities (***r*** = 0.298,0.105;***P***<0.01), and self-efficacy was significantly and positively correlated with emotional management abilities (***r*** = 0.322, ***P***<0.01). Situational motivation and subjective support under self-efficacy were 0.08 and 0.255, respectively, and the adjusted ***R***^***2***^ was 0.107. Self-efficacy played a fully mediating role between physical activities and emotional management abilities, with a total effect value of 0.032. The values of the direct and indirect effects were 0.003 and 0.029, accounting for 8.95% and 90.74% of the total effect, respectively.

**Conclusion:**

The physical exercise behavior of college students is primarily characterized by low intensity. Physical exercise among college students can positively predict their ability to regulate emotions. Self-efficacy fully mediates the relationship between physical exercise and emotion regulation ability among college students. College students can indirectly influence their ability to regulate emotions through physical exercise and self-efficacy.

## Introduction

The "China National Mental Health Development Report (2021–2022)" indicates that young people are a high-risk group for depression and anxiety, with a risk detection rate of 24.1% for ages 18 to 24, significantly higher than other age groups [1]. A similar trend is observed in the age differences in anxiety risk detection rates. Some scholars believe that college students’ emotion regulation ability and mental health are intrinsically linked. Mental health is the external manifestation of emotion regulation ability, providing essential psychological resources for individual emotional management, including self-efficacy and resilience [2]. Emotion regulation ability is essential to mental health, guiding mental health development. An individual’s emotional state signifies their reaction to the environment and biological dynamic state when adapting to environmental changes [3, 4]. The emotions behind behaviours reflect the outcome of actions and represent a dynamic adaptation factor.

Emotional regulation capability is a psychological characteristic encompassing the ability to recognize, monitor, and influence one’s emotions and identify and respond appropriately to the surrounding context [4, 5]. Emotion regulation theory posits that managing emotions does not involve eliminating or suppressing them but adjusting how emotions are expressed after becoming aware of them [6]. Emotions change physiological activities, subjective experiences, and expressive behaviours through specific strategies and mechanisms. Poor emotional regulation ability can make it difficult for college students to cope with stress in life and learning, thereby increasing the risk of anxiety, depression, and other mental health issues. Prolonged emotional distress may also lead to problems such as sleep disorders and eating disorders [7].

Exercise psychology posits that physical exercise has a significantly positive impact on emotion regulation ability, with sports of various intensities effectively enhancing college students’ ability to regulate emotions [8]. Physical exercise promotes personality traits, cognitive styles, and social support among the youth, aiding in alleviating psychological distress. The Health Belief Model suggests that the perceived threats to health and the evaluation of the benefits of health-related actions determine an individual’s health behaviour [9, 10]. College students of different genders, grades, and age groups show variability in their physical exercise behaviours. Previous scholars have confirmed statistical differences in the frequency, intensity, and duration of sports participation among college students of different genders (*P*<0.01) [11–13]; specifically, males have a higher participation willingness than females, and lower-grade female students have a higher exercise intensity than higher-grade groups. Physiological factors related to males are a major factor causing gender differences in participation in physical exercise behaviours. Regular aerobic exercise can activate the motor centre of the cerebral cortex, replacing involuntary exercise with constructive voluntary exercise to address anxiety and adverse stress [14]. High levels of physical exercise can enhance the function of vascular endothelial cells, promote the release of pro-inflammatory and anti-inflammatory cytokines, reduce the incidence of viral infections, and effectively decrease the risk of suicide [14, 15]. A study indicates that physical exercise plays a significant role in the prevention and treatment of many chronic or age-related diseases (such as musculoskeletal diseases, metabolic diseases, and cardiovascular diseases) [16]. Further studies have indicated that intrinsic motivation for exercise plays a critical role in determining mental health [17, 18]. Among male participants with a higher level of physical activity, the impact of intrinsic motivation on emotional health is substantial. Consequently, this study proposes Hypothesis ***H1*:** Physical exercise can significantly and positively affect college students’ emotional regulation abilities.

Self-efficacy, introduced by social psychologist Albert Bandura, refers to an individual’s belief in their ability to complete a specific task [19]. It encompasses an individual’s capability and confidence in performing specific actions to achieve desired outcomes. Self-efficacy can influence college students’ academic goal-setting, judgment of situations, behavioural choices, and levels of effort [20, 21]. Exercise self-efficacy refers to individuals’ confidence in using their abilities to overcome obstacles when choosing and engaging in physical exercise [22]. The confidence level is related to individuals’ assessments of their abilities in physical exercise; the higher the assessment, the more likely individuals are to participate in and adhere to physical exercise actively [23]. Individuals with high levels of exercise self-efficacy can adapt well to social situations, resolve issues that arise during regular exercise with a high level of confidence, and maintain set exercise goals [24]. Individuals with low exercise self-efficacy are more susceptible to the influence of their peers and social circles. If their peers or social networks need more support or have a negative attitude towards physical exercise, they may be more easily discouraged from continuing [25]. Exercise self-efficacy can regulate emotional states, interpersonal relationships, self-esteem, and other health indicators, giving individuals a sense of life’s meaning [26]. Should individuals lack exercise self-efficacy, they may lose the motivation to continue exercising, feel directionless, and develop psychological issues such as depression and anxiety, leading to a decline in emotional management skills and awareness [27]. Physical exercise can also increase the frequency of interactions among peers, enhance an individual’s interpersonal level, and gradually improve exercise self-efficacy. Individuals with high levels of exercise self-efficacy possess sufficient confidence and capability to deal with adverse situations, proactively integrate into a harmonious exercise environment, experience pleasant emotions during physical exercise, and exhibit more prosocial behaviour [28, 29]. Consequently, this leads to hypothesis ***H2***: Self-efficacy can significantly affect college students’ emotional regulation abilities.

Emotional management in college students encompasses all the responsive behaviours adopted to adapt to campus cultural life [30]. Specifically, it refers to the feedback adopted by college students in response to daily emotional changes in accordance with socially mainstream accepted methods and practices. Emotion Regulation Ability, stemming from the theory of emotional intelligence, is the capacity of individuals to identify, understand, express, and regulate their own emotions [31]. The ability to regulate emotions is related to the complexity and flexibility of the emotions themselves. Moreover, poor emotion regulation ability is often considered to be the foundation of common negative emotional states, such as aggression, anxiety, and depression [32]. Studies have indicated that ADHD and emotional dysregulation may pose interactive risks to an individual’s health [33]. Adolescence represents a dynamic period for the development of emotional regulation abilities, with parents’ emotional socialization processes impacting the monitoring, assessment, and modification of emotional goals within the adolescent population [34]. Effective emotional regulation significantly reduces an individual’s propensity to inhibit behaviours in emotionally charged environments [35]. It enables individuals to swiftly stabilize their mindset in the face of insurmountable challenges, mitigating the direct impact of external negative factors on their emotions. The level of emotional management influences the manner of personal survival and development among college students [36]. Unresolved long-term emotional distress not only diminishes the quality of personal life but also leads to a loss of work enthusiasm, impacts interpersonal relationships, and affects the individual’s standard of living [37]. To adapt to the environment or achieve goals, college students can effectively mitigate the adverse effects of negative emotions on themselves by changing their personality traits and mastering ways to cope with stress and stabilize emotions [38]. College students with high levels of emotional management can remain calm and rational when facing challenges or making decisions. They can consider issues more comprehensively, better avoid the interference of negative emotions in decision-making, and improve the efficiency and quality of problem-solving. Accordingly, this research proposes Hypothesis ***H3*:** Self-efficacy plays a mediating role in the impact of physical exercise on the emotional regulation abilities of college students.

According to social cognitive theory, individuals can form assessments of their capabilities in physical exercise by observing the behaviour and outcomes of others in sports activities [39]. If college students observe their peers actively engaging in physical exercise and achieving positive results, they may enhance their assessments of their abilities in physical exercise [40]. A study indicates that the efficacy of emotion regulation plays a significant role in fostering positive emotions among college students, unlocking their potential, and improving their physical and mental health [41]. Further research indicates that active participation in physical exercise not only directly enhances college students’ levels of subjective well-being but also indirectly affects their life satisfaction by improving their self-efficacy and emotional regulation abilities [42]. This study aims to investigate the relationship between physical exercise and emotional regulation ability, further deepening and advancing research on the impact of self-efficacy on the daily learning and life of college student groups. By analyzing the direct impact of physical exercise on self-efficacy and self-efficacy on emotional regulation ability, this study provides a foundation for subsequent tests of mediating effects.

**Fig 1:**
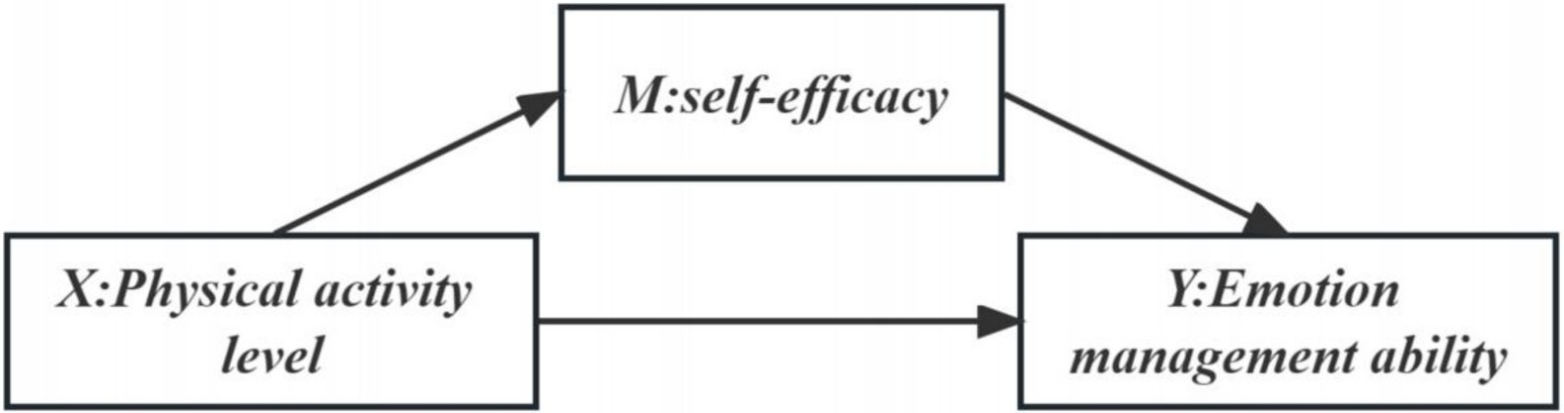
Conceptual model diagram.

Previous studies have shown that there is a positive relationship between physical education and variables such as anxiety and psychological resilience, suggesting that there are also associations between college students’ physical exercise, self-efficacy, and emotion regulation [43]. However, it is still unclear whether self-efficacy can explain the intrinsic mechanism of how physical exercise affects college student’s ability to regulate emotions. Therefore, this study aims to investigate the level of physical exercise, self-efficacy, and emotion regulation abilities among college students to increase social attention and provide intervention strategies and a theoretical basis for improving the emotion regulation abilities of college students by exploring the mediating role of self-efficacy **(The mediating effect is shown in Fig 1)**.

## Materials and methods

### Subjects

By using a stratified random sampling method, a questionnaire survey was conducted in December 2022. The three universities involved in the survey were Nanjing Xiaozhuang University, Tongda College of Nanjing University of Posts and Telecommunications, and Yangzhou Polytechnic Institute. The sample distribution is shown in **Table 1**.

**Table 1:** List of the distribution of research subjects.

Norm		Number of people	Percentage
Distinguishing between			
Gender	Male	2,037	37.5
	Female	3,393	62.5
Grade			
	Freshman	1,772	32.6
	Sophomore	2,825	52.0
	Junior	489	9.0
	Senior	344	6.3
(Grand) Total			
		5,430	100.0

**Fig 2:**
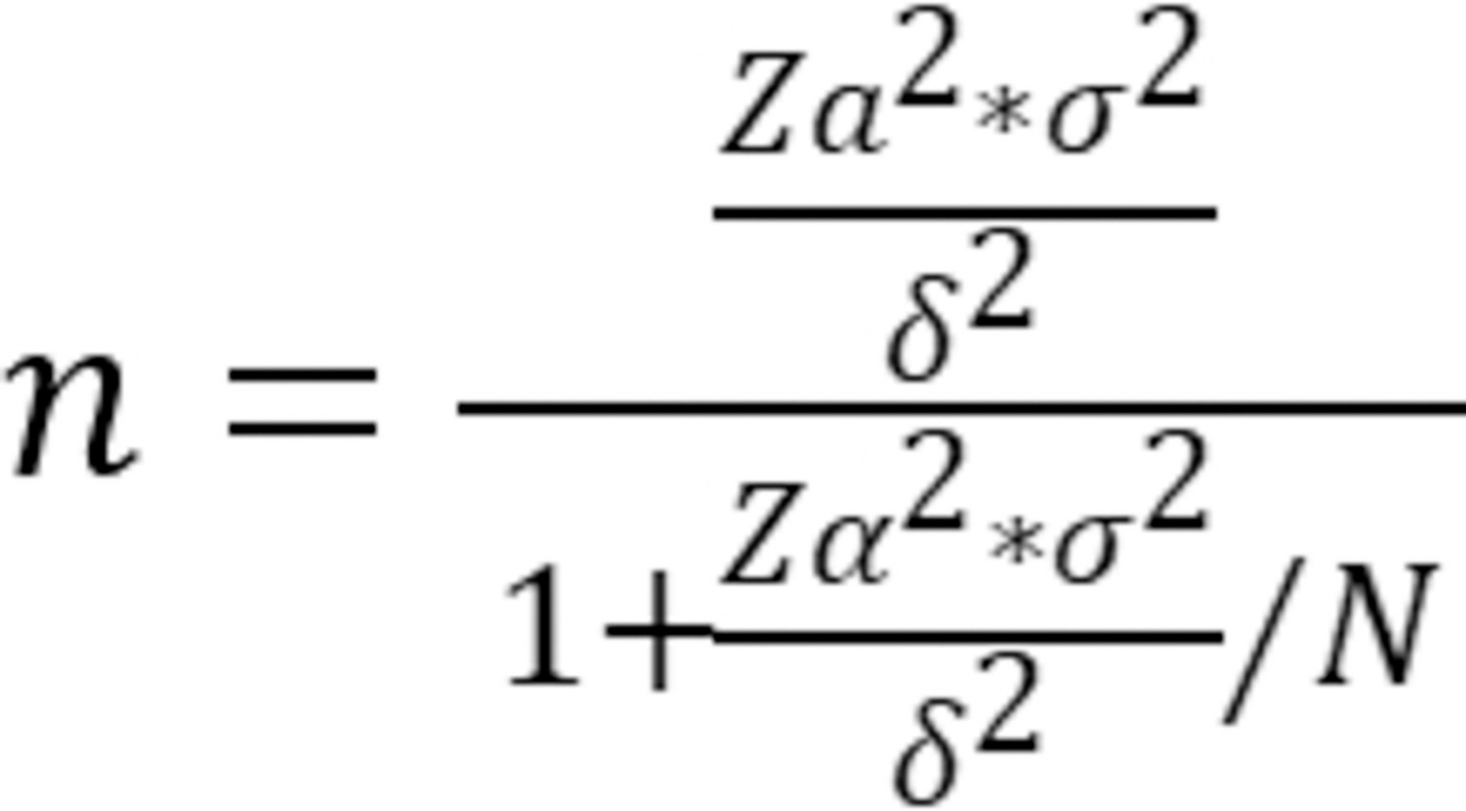
Sample size calculation formula.

The survey was conducted using Questionnaire Star software, yielding 5430 valid responses. The minimum sample size was calculated using **Formula (As shown in Fig 2)**. The parameter (*n*) refers to the required sample size; *N* denotes the total population size (the overall number of individuals in the study); *Zα* represents the *Z*-value from the standard normal distribution corresponding to the significance level *α* (where *Z* typically denotes the quantiles of the normal distribution). *δ* indicates the minimum effect size (effect size) one hopes to detect; generally, the smaller this value, the larger the required sample size. *P* denotes the probability of an event occurring. In various types of studies, it represents the rate of event occurrence under baseline conditions or the proportion in specific populations, etc. *(1-p)* refers to the probability of the event not occurring. Together, (*p*) and *(1-p)* describe the overall distribution of an event. The numerator term *(Zα​/δ)*^*2*^*×p×(1−p)* is used to calculate the required sample size given a specific effect size, event occurrence probability, and confidence level. The denominator term *1+[(Zα​/δ)*^*2*^*×p×(1−p)]/N* is a correction factor used to adjust for the impact of finite population size. When the total population size (*N*) is large, this correction factor approaches 1, reducing its influence on the sample size. With a Type I error (α) set at 0.05, the allowable error (δ) set at 0.01, and the sample rate (ρ) set at 0.05. Upon consulting the official websites of three universities, the total enrollment was found to be 33,761 students (data updated in 2022). Thus, the finite population size N was set to 33,761, resulting in a minimum sample size of 1748.

### Tools

#### Physical Activity Rating Scale (PARS-3)

PARS-3 was compiled by Japanese scholar Hashimoto (1979) and revised by Liang (1994) [44, 45]. The PARS-3 was designed to examine the amount of physical activity in terms of intensity, frequency, and duration of a single exercise session, and measure the level of participation of the subjects. In the specific questionnaire completion, each question item was divided into five levels and scored from 1 to 5. The raw scores from the questionnaire were calculated using the following formula: physical activity score = intensity*time−1. in the scoring structure for PARS-3 is as follows: scores less than or equal to 19 points indicate low exercise involvement, scores between 20 and 42 suggest moderate exercise, and scores 43 and above imply high levels of exercise. The results of PARS-3 represent a measure of the amount of the participants’ physical activities, and the retest reliability of this instrument is 0.82 [46].

#### Physical Activity Self-efficacy Scale (PASS)

The PASS was developed by Jiang et al.(2018) [47]. On the basis of Voskuil’s definition of physical activity self-efficacy, the scale was crafted specifically for the college student population. The questions within the scale reflect the difficult situations that impede the continuation of college students’ physical activity behaviors. The scale particularly evaluates whether college students can still adhere to their original intentions to participate in physical activities despite difficulties and setbacks. Thus, it highlights the beliefs and endogenous motivation of college students about participating in physical activities. Reliability tests show that the Cronbach’s alpha for the total scale and subscales exceeded the acceptable threshold of 0.7. The retest reliability for the total scale, subjective support dimension, and situational motivation dimension was 0.531, 0.359, and 0.562, respectively, and the retest reliabilities of the scales were all good [48].

#### Emotional Intelligence Scale (EIS)

The EIS was developed by Scott et al. based on the theory of Salovey and Mayer (1990) [49, 50]. The Chinese version of the EIS was translated from the scale by Wang Caikang of South China Normal University, which also verified its structural validity (a = 0.83) [51]. Therefore, this study decided to use this scale for investigation and analysis. The scale can be used to assess people’s ability to perceive, understand, express, control, and manage the utilization of their own and others’ emotions. The higher the total score is, the higher the level of individuals’ emotional management abilities will be [52].

### Statistical methods

Statistical analysis of the data was completed using SPSS 26.0. The primary steps in the data analysis are as follows:

The basic demographic information of the research subjects was represented using a frequency distribution table. Chi-square test was used to analyze the differences in physical activity behaviors across different genders and grades. The effect size was used as ***Cramer’s V*** coefficient (***V*** coefficient). According to Clem’s law, a ***V*** coefficient of “0” indicates complete independence, “1” denotes complete correlation, and closer value to “1” implies stronger correlation.

Mean comparison analysis was performed to compare different genders and grades in terms of self-efficacy and emotional management abilities. Descriptive statistics of the specified variables, including mean, standard deviation, F-value, significance, and *η* test results, were compared and reported.

Correlation analysis was used to test the correlations among five indicators: total physical activity rating score, self-efficacy situational motivation, self-efficacy subjective support, total self-efficacy score, and emotional management abilities.

Linear stepwise regression was used to analyze the degree of influence of situational motivation and subjective support. It was also utilized to verify the influence between self-efficacy and emotional management abilities and estimate the value of the mediating effect.

Self-efficacy was used as a mediating variable to test the relationship among physical activity level, self-efficacy, and emotional management abilities.

#### Ethics statement

The study protocol was approved by the ethics committee of Nantong University ((2022(70)). Before formal investigation and testing, the researchers received the informed consent of the subjects involved in this study.

#### Informed consent

We are honored to deliver this questionnaire to you. As the youth of contemporary China, you are the future and hope of our country. We want to take this opportunity to understand your physical fitness and exercise habits. By gathering feedback on your current status, we will develop targeted strategies for promoting the health of college students. The above content serves as the introductory part of the survey. We have obtained informed consent from the participants. If a participant reads the beginning of the questionnaire and is willing to continue, he has voluntarily given his informed consent. If the participant does not wish to complete the questionnaire after reading the beginning of the questionnaire, we will not allow him to continue to fill in the questionnaire and he can choose not to fill it out.

## Results and discussion

### Analysis of descriptive results

Before testing the mechanism of the influence of college students’ physical activities on their emotional management abilities, descriptive statistics were conducted on the main research variables. As shown in Table 2, presents an overview of college students’ physical activities. Of the surveyed 5,430 students, the respondents comprised 2,037 male students and 3,393 female students. The dominant physical activity behavior among the college students involves low amounts of exercise, accounting for 77.6% of the sample. The volume of physical activity is found to be significantly lower in female students than that of males (***V*** = 0.334, ***P***<0.001). Female students engaging in low exercise volumes constitute 87.59%, whereas only 2.86% report high exercise volumes. When examining the grade distribution, a significant difference is observed in the physical activity levels between male and female students per grade (***V*** = 0.052, ***P***<0.001). Comparison of the distribution rates shows that the highest proportion of students engaging in low volume of physical activities is found in the second year (79.86%), the highest percentage of medium exercisers is in the fourth year (15.7%), and the third year has the highest percentage of high volume exercisers (13.29%).

**Table 2:** List of college students’ physical activities.

Norm		Assemble		Gender				Grade							
				Male (n = 2,037)		Female (n = 3,393)		Freshman (n = 1772)		Sophomore (n = 2825)		Junior (n = 489)		Senior (n = 344)	
		n	%	n	%	n	%	N	%	n	%	n	%	n	%
	Low	4,215	77.6	1,243	61.02	2,972	87.59	1,344	75.85	2,256	79.86	358	73.21	257	74.71
	Medium	710	13.1	386	18.95	324	9.55	271	15.29	319	11.29	66	13.5	54	15.7
	High	505	9.3	408	20.03	97	2.86	157	8.91	250	8.85	65	13.29	33	9.59
	χ2			605.302				28.954							
	***p***			<0.001				<0.001							
	***Cramer’s V***			0.334				0.052							

Descriptive statistics were applied to analyze the survey data. As shown in Table 3, significant differences are found in the situational motivation dimension between different genders (***η² = 0*.*022***, ***P***<0.001), with mean and standard deviation recorded as 23.280±5.398 and 21.580±5.464, respectively. This result suggests a weak correlation between male and female college students’ situational motivation. Significant differences are also found in the subjective support dimensions between different genders (***η² = 0*.*025***, ***P***<0.001). The mean and standard deviation are 16.160±3.464 and 14.980±3.626, respectively. This result indicates that subjective support correlation between male and female college students is stronger than their situational motivation. Although this correlation is statistically significant, its practical value seems relatively lacking. The ratio difference in emotional management abilities between different genders is statistically significant (***η² = 0*.*001***, ***P***<0.05), with mean and standard deviation of 29.960±6.781 and 30.320±5.190, respectively, denoting minimal correlation. Assessment of the standards across different grades indicates that first- and second-year students exhibit a high level of self-efficacy. The fluctuations in their scores are comparatively small, suggesting stable changes (***P***<0.001), which are statistically significant.

**Table 3:** Descriptive statistical analysis.

		assemble		Gender				Grade							
				Male (n = 2037)		Female (n = 3393)		Freshman (n = 1772)		Sophomore (n = 2825)		Junior(n = 489)		Senior (n = 344)	
		M	SD	M	SD	M	SD	M	SD	M	SD	M	SD	M	SD
situational motivation															
		22.220	5.500	23.280	5.398	21.580	5.464	22.570	5.200	22.000	5.544	22.310	5.879	22.100	5.998
	***F***			123.993				3.907							
	***p***			<0.001				0.008							
	***η²***			0.022				0.002							
Subjective support															
		15.420	3.612	16.160	3.464	14.980	3.626	15.730	3.423	15.260	3.605	15.420	3.930	15.140	4.024
	***F***			139.219				6.901							
	***p***			<0.001				<0.001							
	***η²***			0.025				0.004							
self-efficacy															
		37.640	8.835	39.440	8.587	36.560	8.807	38.300	8.328	37.270	8.873	37.730	9.547	37.240	9.791
	***F***			138.398				5.235							
	***p***			<0.001				0.001							
	***η²***			0.025				0.003							
Emotional management skills															
		30.190	5.840	29.960	6.781	30.320	5.190	31.040	5.244	29.740	5.999	30.220	6.079	29.360	6.546
	***F***			4.98				20.677							
	***p***			0.026				<0.001							
	***η²***			0.001				0.011							

### Correlation analysis

As shown in Table 4, correlation analysis based on the findings shows that physical activities, situational motivation, subjective support, and self-efficacy are positively and significantly correlated. Emotional management abilities are weakly correlated with situational motivation (***r = 0*.*303***), subjective support (***r = 0*.*325***), and self-efficacy (***r = 0*.*322***).

**Table 4:** Summary of correlation analysis results.

Variant		Physical activities	Situational motivation	Subjective support	Self-efficacy	Emotional management abilities
Physical activities	***r***	1				
Situational motivation	***r***	0.286^**^	1			
Subjective support	***r***	0.295^**^	0.875^**^	1		
Self-efficacy	***r***	0.298^**^	0.980^**^	0.953^**^	1	
Emotional management abilities	***r***	0.105^**^	0.303^**^	0.325^**^	0.322^**^	1

Note:

** in the text represents ***P***<0.01.

### Multiple stepwise regression analysis

As shown in Tables 5 and 6, the multiple linear regression results, accounting for gender, age, grade, and other control variables, reveal the influence of the independent variables, situational motivation, and subjective support, on the dependent variable, emotional management abilities. As shown in Model 2, adjusted ***R***^***2***^ = 0.444, which indicates that the variables for the explanatory degree of the dependent variable is 44.4%. The appropriateness of the linear regression model implemented in this study is supported by the ANOVA results (***F*** = 123.95, ***p***<0.001). The coefficient test results reveal standardized coefficients of ***β*** = 0.088 and 0.257 for situational motivation and subjective support, respectively, with significance of ***P***<0.001. Thus, situational motivation and subjective support have a significantly positive effect on emotional management abilities, supporting ***H*2**. Moreover, the comparison results of the standardized regression coefficient indicate that the ***β***-value of subjective support (0.257) is greater than that of situational motivation (0.088). This result suggests that subjective support has a greater influence than situational motivation on emotional management abilities.

**Table 5:** Analysis of factors influencing self-efficacy and emotional management abilities.

Variant	β	t
Situational motivation	0.08	3.017***
Subjective support	0.255	9.647***
Adjusted R^2^	0.107	
F-value	326.38***	

Note:

*** ian the text represents ***P***<0.001.

**Table 6:** Regression analysis of impact factors with control variables (excluding confounding factors).

Variant	Model 1		Model 2	
	β	t	β	t
Distinguishing between gender	0.022	1.654	0.077	5.907
Grade	−0.04	−2.011*	−0.018	−0.944
Under 19	0.07	2.441**	0.082	3.03**
19 to 20 years old	0.004	0.147	0.025	1.111
20 years and over	0			
Situational motivation			0.088	3.362***
Subjective support			0.257	9.751***
Adjusted R^2^	0.009		0.444	
F-value	13.994***		123.95***	

Note:

*** in the text represents ***P***<0.001.

### Tests for mediating effects

As shown in Table 7, this study utilizes Model 4 from the Process plugin for mediating effect analysis. According to our analysis in ***Model 1***, the total effect of the independent variable on the dependent variable is significant, confirming Hypothesis ***H1***. Upon further examination of the second model, the effect of the independent variable on the mediating variable is also significant. In the testing of the third model, the effect of the independent and mediating variables on the dependent variable is insignificant. Therefore, according to the causal step approach of mediating effect testing, self-efficacy serves a particular utility in the impact of the independent variable on the dependent variable and acts as a complete mediator, thus confirming Hypothesis ***H3***.

**Table 7:** Mediating effect test.

Model	Model 1		Model 2		Model 3	
Implicit variable	Emotional management abilities		Self-efficacy		Emotional management abilities	
Norm	***β***	***t***	***β***	***t***	***β***	***t***
Physical activities	0.0324	7.75***	0.1396	23.02***	0.0029	0.7042
Self-efficacy					0.2109	23.70***
R^2^	0.1046					
Adjusted R^2^	0.011					
***F***	60.10***					

Note:

*** in the text represents ***P***<0.001.

Refer to Table 8 for the following results; according to the results of the ***bootstrap*** technique, the values of the total effect, direct effect, and indirect effect are 0.0324, 0.0029, and 0.0294, respectively. Hence, in the mediation effect test of self-efficacy, the total and indirect effects are valid, and the direct effect is invalid. Therefore, self-efficacy plays the role of a full mediator in the model, and the indirect effect accounts for 90.74% of the mediation effect in the model. The indirect effect accounts for 90.74% of the total effect. Self-efficacy plays 8.95% of the role in emotional management abilities.

**Table 8:** Results of mediation effect using *bootstrap* technology.

Dependency	Efficiency value	*P*	*LLCI*	*ULCI*	Efficiency ratio
Aggregate effect	0.0324	<0.001	0.0242	0.0406	
Direct effect	0.0029	0.4814	-0.0052	0.0111	8.95%
Indirect effect	0.0294	0.0331	0.0259	0.0331	90.74%

## Discussion

This study examines the role of self-efficacy in the impact of physical exercise on the emotional regulation abilities of Chinese college students. It explores the relationship between physical exercise and college students’ emotional regulation abilities using scales, with Hypotheses 1, 2, and 3 all being validated. Physical exercise can influence college students’ emotional regulation abilities through self-efficacy. Additionally, this study suggests potential benefits to emotional health when college student groups actively participate in physical activities. Most importantly, self-efficacy is key in regulating college students’ daily emotions.

Moreover, exercising self-efficacy can positively affect the persistence of college students’ exercise routines, focus on the exercise process, and effectively identify and overcome challenges and obstacles during exercise. This also highlights the importance of college students being able to screen and evaluate their daily routines. By comprehensively considering the association among physical exercise, self-efficacy, and emotional regulation abilities, scientific evidence can be provided for designing comprehensive intervention plans to promote the psychological health of college students.

### Physical exercise has a significant positive impact on college students’ emotional regulation abilities

Previous studies have shown it is speculated that the mechanism of action may be that physical exercise promotes the release of various neurotransmitters such as endorphins and dopamine in the brain, stimulates cognitive and emotional cognition, and reduces negative emotions such as anxiety, depression, and stress in college students [53, 54]. Research indicates that the level of physical exercise is a crucial indicator affecting the emotional regulation abilities of college students and a means for individuals to accumulate psychological resilience resources [55, 56]. More than six weeks of moderate-intensity physical exercise helps college students overcome more physical and psychological obstacles, release accumulated negative energy, and rid themselves of the troubles caused by adverse emotions [57]. Han Beining’s study found that college students who engage in moderate to high levels of physical activity have significantly better lifestyles, emotional management, and quality of life than those with lower levels of physical activity [58]. Physical exercise is a form of social interaction, especially team sports, which can significantly improve interpersonal relationships. Enhanced interpersonal relationships can, in turn, boost college students’ self-efficacy.

### The positive impact of self-efficacy on college students’ emotional regulation abilities

Self-efficacy refers to an individual’s confidence in their ability to execute a task or behavior successfully. The exercise self-efficacy that college students possess during physical exercise means that specific sports activities can significantly positively affect an individual’s confidence in completing tasks and achieving goals. Research indicates that self-efficacy is influenced by trait anxiety, problem-solving, emotional expression, and social withdrawal [59]. In the daily academic performance of college students, self-management skills can significantly positively impact their self-efficacy [60]. A study has shown that a higher level of interpersonal interaction is associated with reduced negative emotions, improved sleep quality, and increased self-efficacy in real life. In contrast, a higher level of online interpersonal interaction is related to higher negative emotions and poorer sleep quality [61]. Relevant studies indicate that self-efficacy and emotional stability in young populations can buffer the psychological and physical impacts of Adverse Childhood Experiences (ACEs) on adolescents in adulthood [62]. Additionally, research demonstrates that self-efficacy plays a crucial mediating role in helping college students understand and complete stressful tasks [63]. In exerting its effect, self-efficacy involves an individual’s cognition and evaluation of their abilities and the internalization of successful experiences. Positive emotions toward work can simultaneously predict an individual’s self-efficacy and job satisfaction [60]. Concurrently, some scholars believe that self-efficacy positively and constructively influences emotional regulation abilities. High self-efficacy can enhance the function of the prefrontal cortex, improving an individual’s cognitive control over emotional responses, enabling a more rational assessment of situations and adopting appropriate coping strategies when faced with emotional challenges [64]. Self-efficacy may modulate the activity of the amygdala, reducing the overreaction to threat information and thereby mitigating the harm of negative emotions to the organism [65]. According to Achievement Motivation Theory, individuals with high self-efficacy may experience stronger feelings of achievement and satisfaction when completing tasks or facing challenges, thereby promoting the release of serotonin and dopamine and improving emotional states [66]. One of the fundamental functions of self-efficacy is to regulate an individual’s emotional state. According to Self-Determination Theory, within the context of exercise and sports activities, autonomy, competence, and relatedness are closely associated with self-efficacy [67]. Individuals with high self-efficacy can optimize their cognitive processes, channeling adverse emotions that arise during life, study in a stable emotional state with a positive attitude and reasonable coping methods, and promptly adjust their behavior.

### The impact of situational motivation and perceived support on emotional regulation abilities

Specific environments or situations trigger situational motivation. Situational motivation focuses on an individual’s motivational state in a specific context, which can be either positive or negative [68]. Positive situational motivation can inspire a subjective solid desire in individuals to engage in an activity. In contrast, negative situational motivation may affect an individual’s emotional state balance to avoid certain behaviors [69]. An individual’s positive emotions and situational motivation are beneficial for stabilizing emotional states in daily life and learning [70]. Positive thinking among college students can enhance their situational academic motivation and academic commitment. Research by scholars such as Cao shows a significant positive correlation between situational motivation and problem awareness [71]. When individuals face difficulties and challenges, their motivation to solve problems increases accordingly. The pursuit of achievement and the tendency to avoid failure significantly influence their motivation to solve problems, a finding that aligns with problem-solving situational theory [69, 72]. The daily learning of college students is influenced by their environment and specific states, with a high level of situational motivation significantly enhancing their enthusiasm for information-seeking and problem-sharing [73]. Perceived support typically refers to the support an individual perceives from others, including emotional support, informational support, and tangible support [74]. According to achievement motivation theory, perceived support can enhance an individual’s agency, making the college student population feel encouraged and supported, thereby making it more likely for them to pursue and achieve their set goals [75]. Research indicates that when college students receive care from others in specific situations, the interaction between external motivation and intrinsic drive can significantly enrich their subjective experience, thereby effectively enhancing their ability to perform tasks and face challenges [76]. Additionally, studies have shown that perceived support, as a form of social support for an individual’s intrinsic emotional needs, has a direct impact on the psychological development of college students [77]. By fulfilling the individual’s need to be respected, perceived support significantly boosts college students’ confidence and self-esteem. This support from friends, mentors, colleagues, and relevant social organizations not only increases college students’ satisfaction with their emotional experiences but also promotes positive communication and understanding among students.

### The mediating role of self-efficacy in the impact of physical exercise on college students’ emotional regulation abilities

Physical exercise can significantly influence the enhancement of self-efficacy among college students, and the elevation of self-efficacy, in turn, can further encourage them to adopt a rational and mature attitude, engage in reasoned behavior to solve problems and reduce the occurrence of negative emotions such as anxiety and depression. The level of physical exercise and the promotion of self-efficacy are necessary, but more conditions are needed to enhance emotional regulation abilities [78]. N Gutiérrez Ángel and others’ research on college student groups found a positive correlation between physical exercise and self-efficacy [79]. Physical exercise can enhance individuals’ confidence and psychological resilience in facing social challenges, and it also highlights the significant correlation between emotional clarity and emotion regulation, cognitive anxiety, and somatic anxiety. The athletic performance of college students is influenced by various factors, including emotions, highlighting the importance of incorporating emotional components into the realm of sports [80]. Research indicates that among emotion-focused strategies, physical exercise is one of the most successful, serving as a reliable method to prevent depression [81]. Long-term exercise is a significant counterbalance to the emergence of human anxiety. The mood-improving effects of appropriate exercise are attributed to its considerable role in reducing stress and promoting the secretion of neurotransmitters such as endorphins [82]. Additional research, from the perspectives of positive psychology and systemic psychotherapy, explores strategies for enhancing college students’ inherent potential and strengths [83]. It finds that emotional understanding and application are significantly more straightforward to improve and accept than emotional awareness, regulation, and expression. According to Goal Setting Theory, challenging goals can enhance an individual’s motivational drive and performance [84]. An individual’s exercise self-efficacy may affect the level of commitment of college students to their goals. If they believe they can complete a task, they may set higher goals and be more inclined to persist and achieve those goals through relentless effort and resilience [85]. From the perspective of coping strategies, self-efficacy influences the judgments and choices made by college student groups in managing emotional situations, types of emotions, and the intensity of emotions. Individuals with high self-efficacy are more likely to adopt proactive coping strategies rather than passive acceptance strategies [86]. Additionally, in some cases, self-efficacy may only serve as a partial mediator, or not at all, depending on other variables such as the difficulty of the assigned task, environmental factors, and competitive pressures.

## Limitations

Self-reporting allows for the collection of participants’ subjective emotions and perspectives and offers a prompt and convenient means of conveying study findings. However, it is important to acknowledge that the reported data may be subject to response bias and influenced by individual cognitive abilities and emotional states to some degree. Moreover, cross-sectional studies do not establish precise causal relationships, necessitating researchers to augment the sample size and integrate current advancements in big data technology to develop more effective interventions for enhancing emotional management among college students, thereby enhancing the efficacy of the study.

## Conclusions

This study conducted an empirical investigation into the relationship between college students’ levels of physical exercise, self-efficacy, and emotional regulation abilities. Based on previous academic literature on emotional regulation abilities, this study collected data from various universities to propose and test hypotheses. The results show that physical exercise has a positive predictive effect on college students’ self-efficacy and emotional regulation abilities. Upon examination, self-efficacy fully mediates the relationship between college students’ physical exercise and emotional regulation abilities, meaning that college students can indirectly influence their emotional regulation abilities through physical exercise combined with self-efficacy. Additionally, after controlling for gender, age, and grade level, the independent variable still has a significant positive effect on the dependent variable.

The results of this study add new knowledge to the existing literature on emotional regulation abilities. This study timely demonstrates that, against the backdrop of cultivating applied talents for modernization in China, higher education institutions should prioritize the formulation of physical exercise implementation rules, providing a theoretical basis and practical guidance for college students’ emotional health education and physical education practices. This study enriches and expands the self-efficacy theory while also encouraging higher education institutions and related educational organizations to fully consider the practical significance of exercise self-efficacy for college students’ psychological health literacy when intervening in college students’ emotional regulation abilities as part of strengthening school physical education efforts, paving the way for new thinking and methods in college students’ mental health education work.

## Supporting information

S1 Data Survey report on university students’ health and lifestyle habits. (XLSX)

## Abbreviations

PARS-3: Physical Activity Rating Scale
PASS: Physical Activity Self-efficacy Scale
EIS: Emotional Intelligence Scale
SD: Standardized deviation
Boot LLCI: Lower limits of 95% Bootstrap confidence interval
Boot ULCI: Upper limits of 95% Bootstrap confidence interval
